# Unmasking Secondary Hypertension: Renal Artery Stenosis Concealing the Diagnosis of Primary Hyperaldosteronism

**DOI:** 10.7759/cureus.96159

**Published:** 2025-11-05

**Authors:** Paul J Wurtz, Franchesca Farris Cosme, Gordon Macy, Devin Moore, Alexandra Stewart

**Affiliations:** 1 Internal Medicine, Brooke Army Medical Center, San Antonio, USA

**Keywords:** adrenal adenoma, adrenal venous sampling, fibromuscular dysplasia, hypertension, hypokalemia, primary hyperaldosteronism, renal artery stenosis, resistant hypertension, secondary hypertension, spironolactone

## Abstract

A 24-year-old woman with no prior comorbidities presented with severe, treatment-resistant hypertension characterized by episodic headaches and persistent systolic blood pressures above 140 mm Hg despite lifestyle modifications. Initial evaluation revealed hypokalemia and metabolic alkalosis with an aldosterone-to-renin ratio suggestive, but not diagnostic, of primary hyperaldosteronism. Secondary workup excluded pheochromocytoma, Cushing’s syndrome, and thyroid disease. Imaging identified right renal artery fibromuscular dysplasia with angiographic narrowing of approximately 30%. Despite maximal medical therapy, her hypertension persisted, and repeat studies later demonstrated progression of stenosis to 70-75%, prompting angioplasty. The intervention improved arterial patency but failed to normalize her blood pressure or correct her tendency toward hypokalemia.

Re-evaluation demonstrated a 1.7-cm left adrenal mass, and adrenal venous sampling confirmed unilateral aldosterone hypersecretion. The patient underwent laparoscopic adrenalectomy with pathology confirming an aldosterone-producing adenoma. Following surgery, she achieved normalization of potassium and sustained normotension without antihypertensive medications.

This case highlights the diagnostic challenges when two secondary causes of hypertension coexist. Fibromuscular dysplasia initially diverted attention from primary hyperaldosteronism, delaying definitive diagnosis and curative treatment. Persistently suppressed renin and unexplained hypokalemia in the setting of uncontrolled hypertension, even after renal revascularization, should prompt clinicians to revisit the possibility of primary aldosteronism. This case underscores the importance of avoiding premature closure, maintaining vigilance for multiple etiologies, and reassessing the differential diagnosis when treatment of a plausible condition does not yield the expected clinical improvement.

## Introduction

Severe hypertension in a young adult should immediately prompt evaluation for secondary causes, as these are present in up to 10% of hypertensive patients under 40 years of age [1]. Among secondary etiologies, primary hyperaldosteronism (PA) and renovascular disease are among the most clinically relevant and potentially curable. Obstructive sleep apnea (OSA) is another exceedingly common contributor that should be excluded in all patient populations [1]. A careful assessment - including a detailed history for OSA symptoms, targeted laboratory testing, appropriate imaging, and a review of potential medication contributors (e.g., contraceptives, nonsteroidal anti-inflammatory drugs (NSAIDs)) - is warranted to pinpoint the cause of severe hypertension.

Primary aldosteronism, classically characterized by hypertension, hypokalemia, and metabolic alkalosis, accounts for approximately 5-10% of all hypertension cases and up to 20% of resistant hypertension [1,2]. Fibromuscular dysplasia (FMD), a non-atherosclerotic, non-inflammatory arteriopathy that predominantly affects women aged 15-50 years, represents the leading cause of renovascular hypertension in this demographic, with an estimated prevalence of 3-4% among young hypertensive women [1].

Although PA and FMD are each well-recognized causes of secondary hypertension, their coexistence can obscure the diagnostic picture. FMD may be discovered incidentally on imaging and can anchor the diagnostic process, delaying recognition of concurrent endocrine etiologies. Conversely, mild or “borderline” biochemical findings of aldosterone excess may be overlooked when a structural vascular abnormality is present. Such overlap highlights the diagnostic complexity emphasized in the 2025 American College of Cardiology/American Heart Association (ACC/AHA) guidelines on the management of hypertension, which stress re-evaluation when blood pressure remains uncontrolled despite treatment of an identified secondary cause [3].

This case illustrates the challenge of dual pathology in secondary hypertension - a young woman in whom right renal artery FMD initially masked an aldosterone-producing adrenal adenoma. It underscores the importance of maintaining diagnostic vigilance and re-evaluating apparent “partial successes” when clinical improvement does not align with expectations.

## Case presentation

A 24-year-old woman with no significant medical or family history presented with hypertension and episodic severe exertional headaches, occasionally accompanied by vomiting. During one episode, her blood pressure exceeded 200/100 mm Hg. Between episodes, her blood pressure remained persistently elevated in the 140-150/90 mm Hg range despite dietary sodium restriction and regular physical activity. The patient was not taking any prescription or over-the-counter medications including contraceptives. She had a normal body mass index (BMI) and did not smoke or use alcohol/drugs. In her review of systems, she reported a single episode of heart palpitations but denied flushing or sweating. She was started on lisinopril 10 mg in the outpatient setting.

On initial evaluation, her blood pressure was 152/96 mm Hg. Laboratory studies revealed a serum potassium level of 3.3 mmol/L and a bicarbonate of 30 mmol/L, consistent with metabolic alkalosis. Her seated morning plasma aldosterone concentration (PAC) was 16 ng/dL (normal <16 ng/dL) and the plasma renin activity (PRA) was 0.44 ng/mL/hour, yielding an aldosterone-to-renin ratio (ARR) of approximately 36. Subsequent values varied while on angiotensin-converting enzyme inhibitors (ACE-I)/calcium-channel blocker (CCB) therapy (Table 1). No abnormalities were noted on metabolic panels aside from the hypokalemia and metabolic alkalosis. Physical examination at that time did not reveal any abdominal bruits, skin stigmata of Cushing’s syndrome, or thyroid enlargement. 

**Table 1:** Serial plasma aldosterone concentration (PAC), plasma renin activity (PRA), and aldosterone-to-renin ratio (ARR) with concurrent antihypertensive therapy (2020–2023). *Potassium 3.3 mmol/L. **After adrenalectomy. Note: Reference intervals are institution-specific (values shown reflect this laboratory: PAC 0–30 ng/dL; PRA 0.167–5.380 ng/mL/hr; ARR 0.0–30.0).

Date	PAC (ng/dL)	PRA (ng/mL/hr)	ARR	Medication
Reference range	0–30	0.167–5.380	0.0–30.0	—
Day 1	16.1	0.442	36.4*	Lisinopril
Day 2	4.2	1.621	2.6	Lisinopril, amlodipine
Day 3	8.5	0.87	9.8	Lisinopril, amlodipine
Day 4	10.7	0.402	26.6	Lisinopril, amlodipine, carvedilol
Day 5	8.3	0.468	17.7	Lisinopril, amlodipine, spironolactone
Day 6	16.3	43.91	0.4	Lisinopril, nifedipine, chlorthalidone, spironolactone
Day 7	33.7	53.079	0.6	Lisinopril, nifedipine, chlorthalidone
Day 8	11.2	7.303	1.5	Lisinopril, nifedipine, chlorthalidone
Day 9	38.2	2.853	13.4	Lisinopril, nifedipine, chlorthalidone
Day 10	23.1	1.783	13	Lisinopril, nifedipine, chlorthalidone
Day 11	40.5	3.025	13.4	Lisinopril, nifedipine, chlorthalidone
Day 12	2.4	3.118	0.8	None**

Given the borderline ARR, her providers opted for a broad evaluation for secondary hypertension outside of primary aldosteronism. Tests for pheochromocytoma, Cushing’s, and thyroid dysfunction were all within normal limits. Screening for uncommon forms of inherited hypertension (e.g. 11β-hydroxylase deficiency, Liddle’s syndrome) was also unrevealing. Given the patient’s age and sex, renovascular hypertension remained a strong consideration. She was referred for imaging studies of the renal arteries.

**Figure 1:**
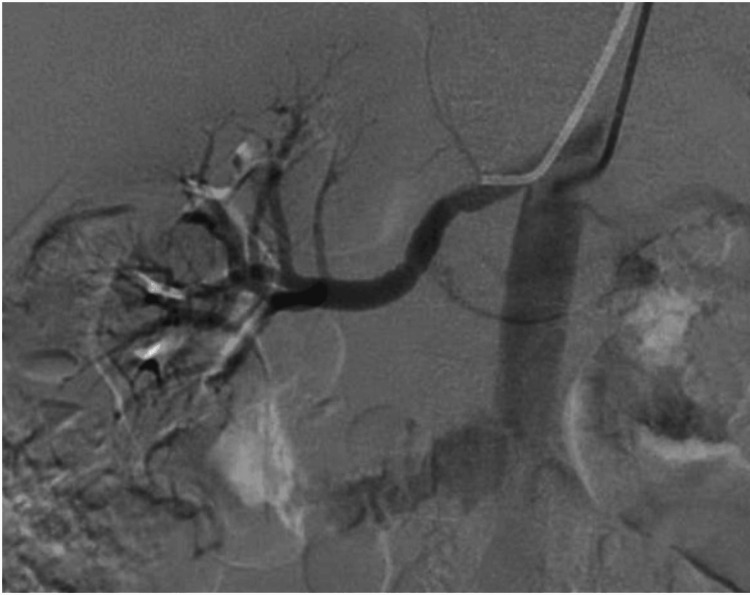
Right renal artery digital-subtraction angiography (DSA). Selective DSA demonstrates subtle multifocal luminal irregularity with a beaded appearance of the mid-segment of the right renal artery, consistent with fibromuscular dysplasia. No critical narrowing is present on this study; the lesion later progressed and was treated with balloon angioplasty.

Duplex ultrasonography of the renal arteries showed an increased velocity in the right renal artery, with a high renal-aortic velocity ratio, findings that were suggestive of significant right renal artery stenosis (RAS). This result prompted confirmatory imaging. Magnetic resonance angiography (MRA) of the renal arteries demonstrated a focal narrowing of approximately 40% in the mid portion of the right renal artery, while the left renal artery appeared normal. A captopril-enhanced renal scan showed symmetric uptake and excretion of tracer by both kidneys, with no delay on the right side to indicate a hemodynamically significant stenosis. An invasive digital-subtraction angiogram (DSA) of the renal arteries was performed (Figure 1).

Access was obtained via femoral approach; the angiogram revealed subtle beading of the mid-right renal artery consistent with FMD, but no critical narrowing. Trans-lesional pressure gradients were not obtained. The lumen was estimated to be narrowed by about 30% at its most severe point. The left renal artery had a normal appearance and caliber. No intervention was performed at that time. In light of the FMD diagnosis, the patient underwent screening for other manifestations of the disease; a cerebral angiogram showed no aneurysms or cerebrovascular abnormalities. 

Over the following year, the patient’s hypertension persisted. Her antihypertensive regimen was intensified to include lisinopril (up-titrated to 30 mg daily), extended-release nifedipine (30 mg daily), chlorthalidone (25 mg daily), and spironolactone (100 mg daily), which was initiated as a fourth-line option. Despite this, her blood pressure remained approximately 150/90 mm Hg. Fourteen months after the initial angiogram, a repeat renal duplex ultrasound suggested progression of the right renal artery lesion (now with velocity criteria consistent with >60% stenosis). Repeat angiography in August 2022 confirmed approximately 70-75% stenosis of the proximal right renal artery. The lesion was dilated with balloon angioplasty, resulting in <10% residual stenosis. 

Two months after angioplasty, her blood pressure was still above 140/90 mm Hg on the same regimen. Follow-up labs continued to show a tendency toward hypokalemia (serum potassium 3.4 mmol/L), and longitudinal PAC/PRA/ARR trends did not normalize. Given variability in noninvasive imaging and the evolving biochemical profile, we summarized serial PAC/PRA/ARR measurements alongside concurrent antihypertensives to provide context for test interpretation (Table 1). At this point, the patient sought a second opinion. The consulting specialist revisited the possibility of primary hyperaldosteronism, pointing out that the patient’s initial presentation with hypertension was accompanied by unexplained hypokalemia and metabolic alkalosis - features more consistent with primary aldosteronism than with isolated FMD. The theory was that the underlying primary aldosteronism might have been masked by the coexisting RAS.

**Figure 2:**
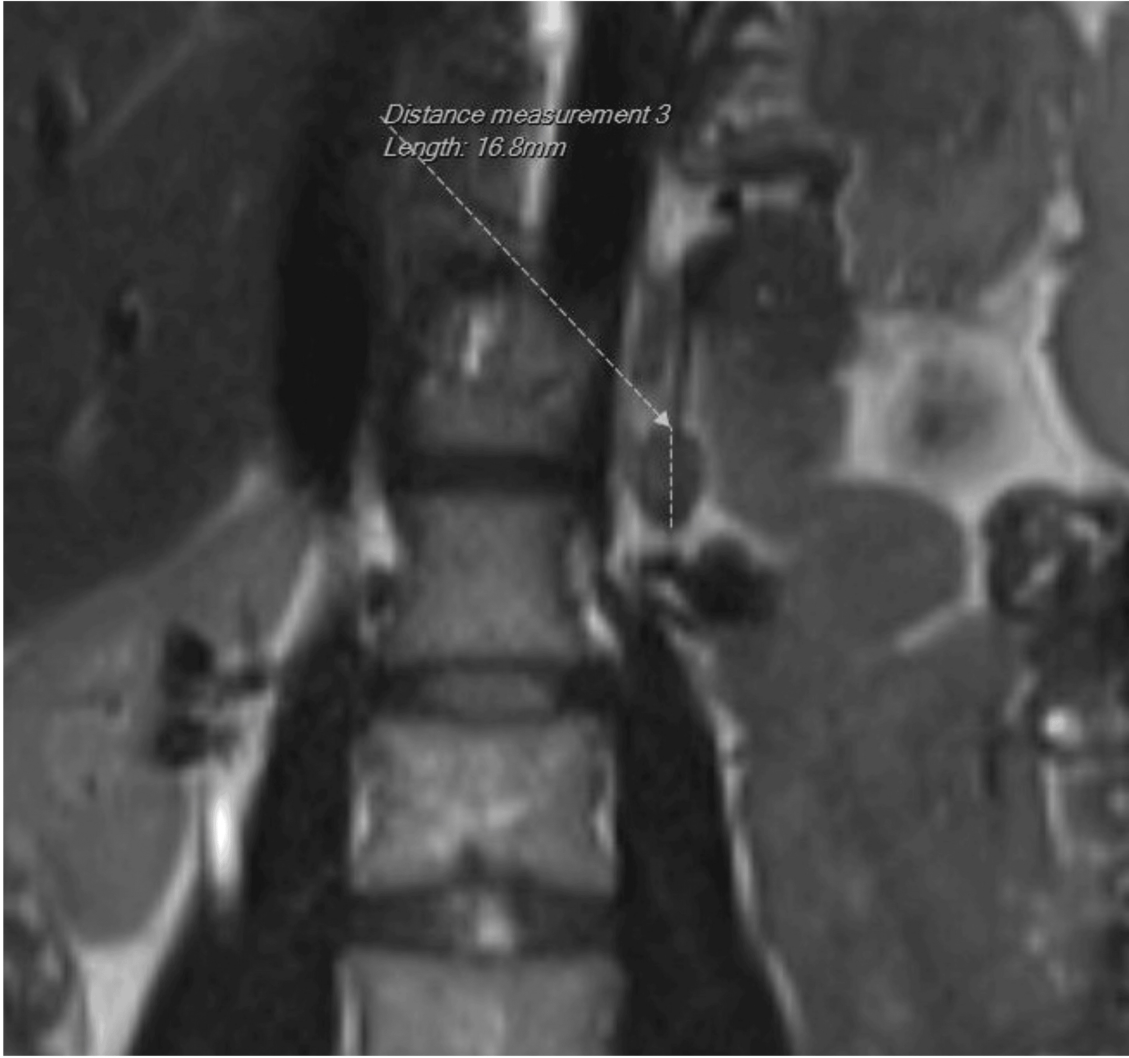
Left adrenal adenoma on MRI. Coronal adrenal-protocol magnetic resonance imaging (MRI) shows a 1.7-cm nodule in the left adrenal gland (dashed caliper, measured 16.8 mm). Adrenal venous sampling subsequently lateralized aldosterone secretion to the left, consistent with an aldosterone-producing adenoma.

An MRI of the adrenal glands revealed a 1.7-cm mass in the left adrenal gland, consistent with an adrenal adenoma (Figure 2).

To confirm that this adenoma was responsible for aldosterone overproduction, adrenal venous sampling (AVS) was performed, which showed lateralized aldosterone secretion from the left side, establishing the diagnosis of primary aldosteronism due to an aldosterone-producing adrenal adenoma.

The patient underwent a left adrenalectomy. Pathology confirmed an adrenal cortical adenoma. Within one month, her serum potassium normalized to 4.2 mmol/L and her blood pressure was consistently in the range of 120-130/80 mm Hg. She was able to discontinue all antihypertensive medications. At a follow-up visit three months post-operatively, she remained normotensive and symptom-free. A comprehensive timeline of PAC, PRA, ARR, and concurrent medications is provided (Table 1).

## Discussion

The combination of spontaneous hypokalemia and metabolic alkalosis in the context of resistant hypertension is strongly suggestive of primary hyperaldosteronism, but this trio is also seen in Liddle’s syndrome, although in that diagnosis, both renin and aldosterone are suppressed [2]. An ARR of 36 is above the usual screening cutoff for primary aldosteronism, although the plasma aldosterone level of 16 ng/dL is only modestly elevated and the renin, while low, is not fully suppressed. In a typical case of primary aldosteronism, one might see a higher aldosterone level (often >20 ng/dL) with an undetectable renin [4]. This patient’s results fall into a gray zone - they raise suspicion but are not diagnostic. It is possible that external factors (e.g. lisinopril or dietary sodium intake) could have influenced these values. Confirmatory testing, such as a saline infusion suppression test, would normally be indicated to definitively diagnose primary hyperaldosteronism. However, these were not performed here, given that her repeat ARR was 2.6 (taken with the patient on lisinopril). Due to the severity, alternative etiologies were considered with the possibility of more than one process at play. Notably, her low renin level is somewhat inconsistent with renovascular hypertension (where one would expect an elevated renin), but it does not rule it out, especially if a concurrent primary aldosteronism is suppressing renin release. At this stage, it was appropriate to further investigate the possibility of primary aldosteronism or pursue a broader workup.

Renovascular hypertension, particularly due to FMD, is a compelling alternative diagnosis in a young woman. FMD typically affects the mid-portion of the renal arteries and can cause severe hypertension [5]. Diagnosis requires specialized imaging, but interpretation is prone to operator-dependent error and variability [6]. The duplex ultrasound flagged a possible high-grade stenosis in the right renal artery, but subsequent MRA suggested only a mild stenosis, and the captopril renogram did not show functional evidence of reduced perfusion or function in the right kidney. Measurement of a trans-lesional pressure gradient can help determine hemodynamic significance of fibromuscular dysplasia lesions, though this was not performed in our case. In a patient with true hemodynamically significant RAS, one might expect the affected kidney to show delayed radiotracer excretion under the ACE-inhibited conditions of a captopril renal scan (due to reduced glomerular filtration from lowered angiotensin II-mediated efferent arteriolar tone). The absence of such findings here called into question the significance of the lesion seen on MRA. Noninvasive tests for RAS each have their limitations: Doppler ultrasound can produce false positives especially in less experienced hands or in unfavorable body habitus; MRA can overestimate stenosis due to resolution limits; and nuclear renography can miss a stenosis that is present but not yet causing significant functional impairment [5-7]. Given these discordant results, the prudent next step was to obtain a definitive anatomical evaluation with renal arteriography, which is an invasive test but the gold standard for diagnosing RAS. This clarifies whether a stenosis is truly present and significant. It’s worth noting that FMD could still be present even with only a moderate narrowing seen on MRA - the characteristic “string-of-beads” appearance might not always correlate with a high grade of stenosis but can still be an important finding [8].

The angiographic findings confirmed FMD in the right renal artery. This diagnosis fit the patient’s demographic and suggested a potential cause for her hypertension. However, the stenosis caused by the FMD appeared mild (only 30%), which complicated the picture. FMD can cause hypertension even with moderate stenosis, possibly through localized changes in renal perfusion and activation of the renin-angiotensin-aldosterone system (RAAS), but one might not expect severe, refractory hypertension from a 30% narrowing alone [5]. The lack of a high-grade lesion at angiography could explain why the prior noninvasive tests were inconsistent. A reasonable approach for mild FMD lesions is medical management with antihypertensive therapy, particularly ACE inhibitors or angiotensin receptor blockers (ARBs), which address the RAAS activation [1], which the patient was already on (lisinopril). Additionally, since FMD can be a systemic arterial disease, screening for other aneurysms was appropriately done [6]. It remained possible that she had concurrent primary aldosteronism, but with the FMD diagnosis made, there was a tendency to attribute all her hypertension to FMD.

The stenosis in the right renal artery progressed from mild to hemodynamically significant. This progression is consistent with FMD, which can worsen over time, leading to more severe narrowing and thus more activation of the RAAS. The patient now had what appeared to be a functionally significant RAS (as evidenced by ultrasound and confirmed by angiography) and underwent an angioplasty. Angioplasty (without stenting) is the preferred treatment for FMD-related RAS and often has a high success rate in improving blood pressure control. Outcomes for blood pressure improvement after FMD angioplasty show that a majority of patients achieve cure or clinically meaningful improvement. Strict hypertension cure (off medications) occurs in around 36-60% of cases depending on definition and patient selection, but improved blood pressure control is reliably achieved in up to 70-90% when including those with reduced medication burden [9]. Technically, the procedure went well, almost completely relieving the stenosis. One would hope to see an improvement in blood pressure after revascularization if that stenosis were the primary driver of her hypertension. Notably, even before angioplasty, the patient was on a comprehensive medical regimen that included spironolactone. Its inclusion may reflect empiric treatment for suspected hyperaldosteronism; however, this is not certain as some experts may suggest its use early on in resistant hypertension regardless of the underlying etiology [10]. While spironolactone can help control blood pressure in both hyperaldosteronism and other forms of resistant hypertension, it can confound aldosterone and renin measurements. After a successful angioplasty, we would expect, if RAS was the sole issue, that the RAAS system drive would diminish and blood pressure would improve, allowing deintensification of medications.

It became apparent that correcting the RAS did not cure her hypertension. This outcome forced reconsideration of earlier assumptions. The persistent hypertension and hypokalemia, despite the resolution of the RAS, strongly suggested that primary hyperaldosteronism was indeed the major driver all along, with FMD playing a smaller role. In retrospect, the initial clues - suppressed renin, even in the context of significant hypertension - aligned more with primary aldosteronism than secondary aldosteronism [3]. Typically, a moderate-to-severe RAS would drive renin up, not down. The fact that her renin was low from the beginning implied that an autonomous source of aldosterone was keeping renin suppressed, which points to an adrenal aldosterone-producing adenoma or bilateral adrenal hyperplasia. The progression of the RAS and its treatment may have clouded the picture, especially since the team’s focus understandably shifted to the striking angiographic findings. This illustrates how anchoring on one abnormality can lead to overlooking a coexisting condition. A fresh perspective helped redirect attention to the unresolved question of aldosteronism.

In the setting of confirmed hyperaldosteronism, a 1.7 cm left adrenal mass on imaging confirmed by AVS strongly suggests an aldosterone-producing adenoma. AVS is the gold standard for determining whether aldosterone excess is coming from one adrenal gland or both. The use of AVS is important because about half of primary aldosteronism cases are due to bilateral adrenal hyperplasia, which would be managed medically rather than surgically [4]. Here, unilateral disease meant the patient was a candidate for curative surgery. It is worth noting how the aldosterone excess could have been initially underestimated. The patient had been on lisinopril for resistant hypertension, which can raise renin levels and lower the aldosterone-to-renin ratio, masking existing primary aldosteronism. Additionally, the concurrent RAS (FMD) might have added a secondary component of hyperreninemia at certain points by hampering the physiological feedback loops.

This case illustrates several crucial lessons in both clinical reasoning and secondary hypertension. It underscores the need to maintain a broad differential diagnosis and a willingness to pursue multiple lines of investigation when confronted with puzzling or refractory conditions. The patient’s initial workup hinted at primary hyperaldosteronism, but that diagnosis was left in diagnostic limbo when imaging suggested FMD/RAS. It is tempting in such situations to anchor on a single abnormal finding and attribute all symptoms to it - a cognitive bias known as “premature closure” [11]. In retrospect, the persistently suppressed renin and hypokalemia were red flags for coexisting primary hyperaldosteronism. At the time, however, her repeat ARR was likely confounded by ACEi use and concomitant FMD with RAS. While FMD-related RAS can lead to secondary hyperaldosteronism, this typically occurs with elevated renin. In her case, the low renin actually pointed to a primary process - an important clue initially obscured by anchoring on renovascular disease. Moreover, the introduction of medications like spironolactone (unable to be held due to dangerously high blood pressures) altered her RAAS feedback, making interpretation of follow-up ARR challenging [12]. Misleading initial tests, whether false negatives or falsely reassuring results, were clarified only through specialist input and deliberate reevaluation. Ultimately, a multidisciplinary perspective was crucial in disentangling overlapping etiologies and avoiding diagnostic anchoring. When blood pressure remains uncontrolled despite addressing one apparent cause, clinicians should revisit the differential diagnosis rather than assuming treatment failure. For this patient, curing her primary hyperaldosteronism was the step that ultimately normalized her blood pressure, while anatomic correction of her renal artery lesion alone was insufficient [13,14].

The diagnostic testing surrounding resistant hypertension is nuanced, and more than one contributing etiology may be present [12]. Contemporary guideline recommendations emphasize structured evaluation, integration of biochemical and imaging data, and multidisciplinary management for patients with secondary hypertension [12]. By systematically addressing all abnormalities and reassessing the patient’s response to each intervention, clinicians can avoid missing the hidden diagnosis and achieve the best outcomes for patients [13,14].

Limitations

This case was limited by the absence of confirmatory suppression testing for primary aldosteronism and the inability to withhold mineralocorticoid receptor antagonists before adrenal venous sampling due to refractory hypertension. Additionally, a trans-lesional pressure gradient was not measured during renal angiography, which may have provided further hemodynamic context.

## Conclusions

The dual pathology in this case teaches caution about one diagnosis overshadowing another. It reinforces the value of thorough investigations and the careful integration of laboratory and imaging data. The major cognitive lesson is to remain vigilant for coexistent conditions, especially when the clinical pieces do not perfectly align with a single explanation. By systematically addressing all abnormalities and reassessing the patient’s response to each intervention, clinicians can avoid missing the hidden diagnosis, thereby achieving the best outcomes for their patients. 

Clinical takeaways include that persistent hypertension despite correction of an apparent secondary cause should prompt re-evaluation for coexisting etiologies. Medications that interfere with the renin-angiotensin-aldosterone system - particularly ACE inhibitors and mineralocorticoid receptor antagonists - can mask primary aldosteronism by altering ARR interpretation. When the clinical picture remains inconsistent or renin is suppressed despite intervention, adrenal venous sampling should be pursued to clarify the source of aldosterone excess.
